# Impact of place of residence on place of death in Wales: an observational study

**DOI:** 10.1186/s12904-017-0261-5

**Published:** 2017-12-12

**Authors:** S. R. Ziwary, D. Samad, C. D. Johnson, R. T. Edwards

**Affiliations:** 1 Centre for Health Economics and Medicines Evaluation (CHEME), Bangor University, Ardudwy Building, Bangor, LL57 2PZ UK; 2 University Malaya, Kuala Lumpur, Malaysia; 3 Speciality registrar in Public Health, Public Health Wales, Mold, UK

**Keywords:** Wales, Deprivation, Death place, Socioeconomic, Residence

## Abstract

**Background:**

Previous research in England showed that deprivation level of a person’s place of residence affects the place of death and quality of care received at the end of life. People dying in their preferred place of death has also been shown to act as an indication for high quality of end of life care services and social equality. This study expands on current research to explore the effects of deprivation and place of residence on health related choices and place of death in Wales.

**Methods:**

We used ten years combined mortality statistics from 2005 to 2014 and Welsh Index of Multiple Deprivation rankings for each lower super output area. After accounting for the population’s age, the number of deaths in Hospital, Hospice, Home, Care Home, Psychiatric Units, and Elsewhere were compared across deprivation quintiles.

**Results:**

Distribution of place of death was found to be concentrated in three places – hospital (60%), home (21%) and care home (13%). Results from this study shows a high number of hospital deaths, especially for more deprived areas, despite being the least preferred place of death.

**Conclusion:**

This is the first Welsh study investigating place of death in relation to deprivation, which could be of major importance to academics, end of life care providers and policy makers interested in to reduce health care inequality in Wales.

## Background

People dying in their preferred place of death acts as an indication of high quality of end of life care services and social equality [1]. A person’s place of residence has been repeatedly shown to be the most preferred place of death for all population groups, including terminally ill patients [2–4], different age groups [3], and residents of urban and rural areas [5] in the United Kingdom (UK). According to a Marie Curie Cancer Care report [6], the preferred places of death for the UK population was home (63%), hospice (28%), hospital (8%) and care home (1%). The registered place of death in the UK showed a different pattern, whereby most people died in hospital (54.8%) followed by home (20.8%), care home (17.8%), hospice (4.5%) and elsewhere (2.1%). This means that more than 70% of the population are dying in the two locations that are least preferred across the country.

This large difference between actual and preferred places of death in the population, especially in terms of high hospital deaths, has been linked to multiple factors. The King’s Fund report from England shows high hospital stays and death are due to patient attributes (such as age, sex and health status), availability of community services, access to hospital services, the way in which hospital services are managed, deprivation and geographical access to a hospital over a more preferred place [7]. Areas that are well-developed and rank lower in deprivation rankings contains integrated home services for older people leading to lower rates of hospital bed use and hospital deaths [6, 7].

A considerable amount of published research has shown the adverse impacts of low socioeconomic status and place of residence with high deprivation on health and health-related choices, such as ‘place of death’ [8–14]. The Marmot Report shows a significant difference exists in the life expectancy of people living in the same city but with different economic status [8]. The report showed that the poor, compared to the better-off, had 17 years of lower life expectancy and disability-free life expectancy. This indicates that the deprived population are not only likely to die sooner, but also are likely to spend a relatively greater portion of their lives with a disability.

England has been a pioneer in studying the effect of deprivation on health and place of death. A research conducted by End of Life Care Intelligence Network explored the role of socioeconomic deprivation, age, gender and cause of death in mortality rate and place of death among the English population [15]. The study showed a correlation between the deprivation level of place of residence and the place of death in England. It compared mortality rates, cause of death and age at time of death with the deprivation quintile to see how deprivation affects these health variables. The study postulated that people from the most deprived quintile were 29% more likely to die in the hospital than those from the least deprived quintile. However, a variety of other factors have also been linked with the place of death such as, the disease or event causing the death, its trajectory or acuteness, social factors affecting access to services (public or private), living arrangements and the availability of family or other local support [15].

While studies exploring the correlation between the deprivation level of place of residence and the location of death have taken place in other countries, such as England, similar research has not been done in Wales. The Welsh government’s annual report on end of life care shows that 56% of the deaths in Wales occurred in NHS hospitals across the country [16]. Moreover, the degree to which people receive palliative care at the end of life in hospital and are treated with dignity, has been seen to be poorer compared to other healthcare settings [17], which raises a serious concern and supports the need for further research.

The principal aim of this study was to explore the places of death within the Welsh population and see how deprivation level of place of residence affects mortality rates and where a person dies. A secondary aim of the study was to explore the impact of the age distribution of the Welsh population on the place of death and their respective mortality rate.

## Methods

### Mortality data

Mortality data were obtained from the Office for National Statistics (ONS) for the years 2005–2014 [18]. Data provided by the ONS were for residents of Wales, and included number of deaths in each of the six palliative care settings (hospital, hospice, home, care home, psychiatric units and elsewhere) and for five deprivation quintiles. Mortality rates used in the study are per 1000 population per year.

To allow comparative analysis of areas’ deprivation, the Welsh Index of Multiple Deprivation 2014 (WIMD) was used [19]. WIMD is used to calculate deprivation in small areas of Wales, and is suitable for comparative measurements of level of deprivation between one area and another [20]. The Index ranks areas in the order of most to least deprived but does not provide for the exact level of deprivation in any area. WIMD is formed from eight standardized domains: income, employment, health, education, access to services, community safety, physical environment and housing.

Mid-year population estimates of lower super output areas (LSOAs) by single year age bands and WIMD (2014) were accessed directly from the ONS website [21]. Lower super output areas (LSOAs) are built from groups of Output Areas used for the 2001 Census (Welsh government, 2014) and are small areas of the country specifically devised to improve the reporting and comparison of local statistics [21]. Both data sets were used to calculate the total population of all 5-year age bands for each LSOA and WIMD quintile. The Office for National Statistics obtains data of deaths from the Civil Registration System which is administered by ONS [22]. To make it consistent with the reference date of mid-year, age at death is adjusted to 30th June, and the key data items used for the aggregation of the provided data consisted of place of death, place of residence, and age.

“Place of death” refers to the physical place of death as shown on the death certificate records, not where the person was cared for at the end of their life. For example, if a care home was actively caring for a person at the end of their life within a hospice setting, the place of death recorded was hospice not care home. The most common places categorized by ONS’ DH1 General Mortality Statistics and used in the study includes: hospital, hospice, home, care home, psychiatric unit and elsewhere. The number of deaths recorded by the ONS did not contain local authority of residence. For these, a local authority was imputed using the distribution of deaths by age and sex known about during the year [22]. Additionally, a small adjustment was made by the ONS for anticipated late registrations to allow for deaths that were not registered at the time the data were extracted. The number of late registrations in the previous year was used as a proxy for late registrations in the current year given the assumption that the number of late registrations does not vary much year to year [22].

### Statistical analysis

Analysis was carried out using indirect standardization (with 95% confidence interval). Descriptive statistics were used to analyse the distribution of populations across 5-year age band and WIMD deprivation quintiles. The mortality rate for each of the six locations (calculated at 5 deprivation quintiles over the ten-year period between 2005 and 2014) for the Welsh population was calculated by adjusting for five-year age bands. Since age-specific rates for each of the WIMD quintiles were not available for the study, the set of mortality rates of each age band from the standard population of Wales were used to calculate standard mortality rates for each of the six locations.

## Results

### Population trends

The average Welsh population in each year between 2005 and 2014 was found to be slightly above 3.1 million people. Distribution of the population was significantly different across age groups, especially above 65. While those aged between 15 to 24 and 40 to 49 were among the groups with the highest population, each containing more than 200,000 people, 85–89 and 90+ groups had the lowest numbers of population, each below 50,000 people. In fact, population numbers declined steadily after 64 for each age band. Age distribution for the Welsh population across all deprivation quintiles was similar to that of the general population. The difference between the number of people aged below 65 (a pivotal age for mortality rates) for each quintile was low, as was for above 65.

### Mortality analysis

**Fig. 1:**
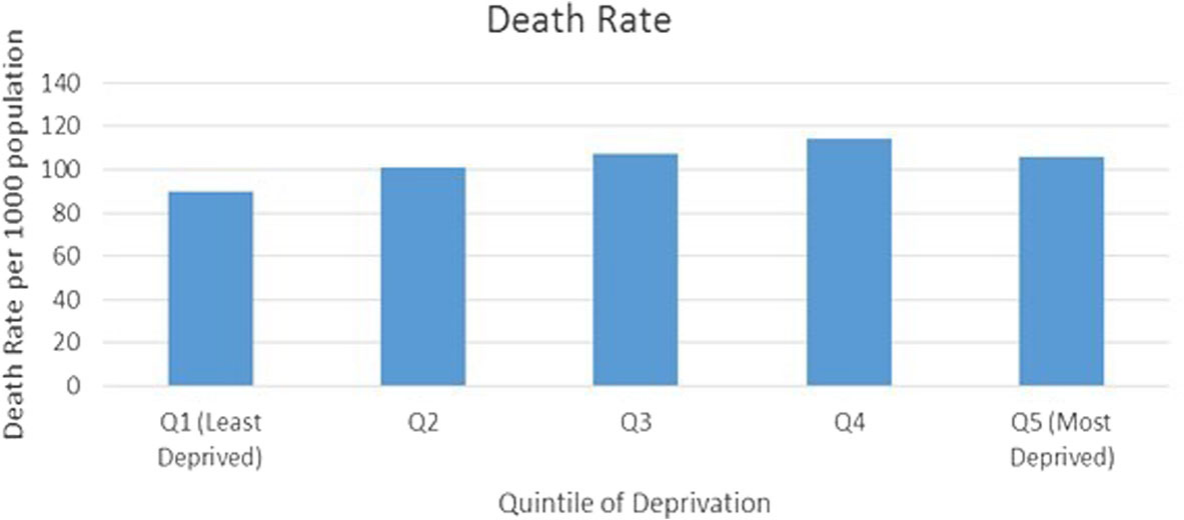
Death rate in each of the deprivation quintile per 1000 population

Figure 1 presents mortality rates for all five WIMD quintiles. The highest mortality rate was seen in quintile 4 (114), followed closely by quintiles 3, 5 and 2 with 107, 106 and 100 respectively. The mortality rate was lowest at 89 in quintile 1, also referred to as the least deprived quintile. As mentioned before, the age distribution for the Welsh population was roughly similar for all deprivation quintiles, hence standardization for age was not necessary.

Aggregation of death figures in all locations showed a progressive increase in deaths as the age-band of population increased. Seventy percent of the deaths among the Welsh population occurred after the age of 70, with the highest numbers seen in the 85–89 year olds (~18%). The majority of deaths recorded over the 10-year period in Wales were found to be in Hospitals (60%), followed by Home (21%), Care Home (13%), Hospice (3%), Elsewhere (2%) and Psychiatric units (< 1%).

**Fig. 2:**
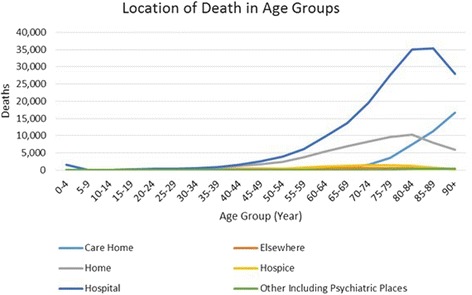
Location of death in all age groups in Wales (2005–2014)

As seen in Fig. 2, there was some variation in the place of death across different age groups. The number of deaths occurring in the hospital and home was seen to rise after the age of 40, care homes after 70s and the rest being steady in other age groups. Hospital was the most common place of death after the age of 40, rising as the age increased. Hospital deaths were recorded at 35,000 on average each year among those aged 80–89, declining after 90. During the 10-year period, the maximum number of deaths at home was seen within the population aged 80–84, where the number was approximately 10,000. In contrast, the number of deaths registered at care homes started increasing late (i.e. after 70s), and surpassed even the number of home deaths from the age of 80 and onwards, reaching 16,000. Hence, while the number of deaths at hospitals and homes decreased after the age of 80, these were replaced by increasing number of deaths in care homes.

After accounting for population age-distribution, the standard mortality rates (SMR) for each of the six location was found to be different across WIMD quintiles. Table 1 shows the SMRs for all five deprivation quintiles (rates with *are significant with 95% confidence interval). Mortality rates in hospitals increased for increasing levels of deprivation (i.e. progressively increases from 82.7 in quintile 1 to 105.4 in quintile 5). Home deaths, like that of hospital deaths, also showed a direct relationship with the level of deprivation (increasing from 79.6 in quintile 1 to 107.1 in quintile 5). In contrast, the SMRs for care home, hospice and psychiatric unit deaths showed no discernible pattern with the increase in deprivation for each quintile, although the rate in Q5 was always lower than the Q1 SMR.

**Table 1:** Age-standardized mortality rates in each of the six locations for WIMD quintiles of Welsh lower super output areas per 1000 population

Place of death	Q1	Q2	Q3	Q4	Q5
Hospital	82.7^b^	92.1^b^	96.6^a^	102.5	105.4^a^
Home	79.6^b^	94.6^a^	104.7	101.6	107.1^a^
Care Home	94.2	116.8^b^	107.2^a^	92.4^a^	75.3^b^
Hospice	127.3^b^	101.6^a^	81.8^a^	87.8	92.9
Psychiatric units	120	58.7^a^	119.3	84.5	95.3
Elsewhere	65.8^b^	98.8	107.3	111.5	115.6

^a^Significant Mortality Rates, *p* ≤ 0.05

^b^Significant Mortality Rates, *p* ≤ 0.001

## Discussion

These new analyses of mortality and place of death within Wales highlight several important issues. The majority of deaths (80%) occurring in the Welsh LSOA populations were among those over 60 years old. The distribution of place of death in the population of the LSOAs of Wales was concentrated in three major places: hospital, home and care home, where 60%, 21%, and 13% of deaths occurred respectively. Hospices, psychiatric units, and elsewhere accounted for less than 6% of deaths altogether. Compared to the general population, the distribution of place of death was seen to differ significantly according to place of residence. Particularly striking was the difference in the probability of dying in hospital or at home for those living in the most deprived LSOAs compared with those in the least deprived LSOAs. The probability of dying in hospital for those living in the least deprived areas was found to be 18% less compared to the general population, whereas those living in the most deprived quintile showed 5% higher probability of dying in hospital compared to the general population. Although previous studies in England had shown an inverse relationship between probability of dying at home and high level of deprivation [1, 23], results from this study shows a direct relationship for Wales.

The difference in the number of deaths seen among the deprivation quintiles were found to be related to the level of deprivation. The least deprived quintile showed the lowest death rate (89 per 1000 population), whereas the highest number of deaths was seen in quintile 4 (114 per 1000 population). In other words, 25 more deaths were registered in quintile 4 compared to quintile 1 per year. When comparing these figures to England, a similar trend can be seen in England. As the age profile was found to be roughly the same in all deprivation quintiles in Wales, the variation in the death rates can be said to be related to the deprivation level rather than the difference in age.

Despite hospitals being the least preferred place of death, findings from this study shows that 60% of the Welsh population still die in hospitals. Comparison across areas with different deprivation rankings using the WIMD showed that the probability of dying in a hospital increased with deprivation. This finding also aligns with the previous studies from the Welsh Government [24], and a population-based study of deaths in hospitals in six European countries including Wales [25]. The mortality statistic report from the Welsh Government shows the effect of access to local service provision on the percentage of deaths in hospital [24]. This rate seems to be higher in areas with less care home and hospice provision. For instance, due to the presence of a hospice run by Marie Curie in Cardiff, some deaths occur in the hospice that would have otherwise occurred in the hospital [16]. A map of hospital sites in Wales show that a large area in the centre and east side of Wales do not have any major hospital, whereas South Wales contains 12 major hospitals [26]. Hence, it could be stated that distance to hospital could be a factor in people dying in other settings.

Overall, 21% of deaths in Wales occur within the home. This number is similar to that seen in England and overall for the UK [17, 23]. However, while looking into distribution of home deaths across deprivation quintiles, the results were contrary to expectations. The number of home deaths increased with the increase in the deprivation level of residence. The SMRs indicated a 21% lower chance of dying at home for those living in the least deprived area and a 7% higher chance of dying at home for those living in the most deprived areas compared to the general population. Given that two-thirds of the population in the UK prefer to die at home [17], results from this study show that people from deprived areas have a better chance of dying in their preferred location in Wales. Although this seems to reflect a positive outcome, it should not be taken at face value without looking into the prevailing circumstances of Wales. These circumstances, which include distance, access to services and cause of death may result in these people’s inability to access health care services rather than having quality health care at home. Although cause of death was not included in this study, previous research from England showed a significant difference in the cause of death among deprivation quintiles [27]. Most common cause of death for the most deprived areas of England was seen to be cancer, while population from the least deprived quintiles died from cardiovascular and respiratory diseases. And also smoking and was seen to be higher in deprived area. This difference in the disease type between the quintile’s might impact chances of home death by increasing sudden deaths in one population compared to another. This requires further exploration.

Care homes were the third most common place of death for the Welsh population (13%). The number of deaths occurring in the least deprived areas was higher compared to deprived areas. Since care and nursing homes have been shown to be the common place of residence for elderly [15] and the majority of deaths in Wales occur in the elderly population, the low rate of care home deaths in the deprived quintiles is a good indicator of the fact that the older generation does not have sufficient access to care homes in these quintiles. England’s report (2010) showed the same results for the English population [23].

Contribution of other places like hospices, psychiatric units and elsewhere as the location of death was very low (˂6%). As death in a hospice is the second most preferred place of death for the population [28], only 3% of Welsh population dying in this setting is another indication of healthcare preferences not being met.

The core strength of the study is that the data for the Welsh population (population estimates, mortality, and deprivation) is not a sample but rather a census from a 10-year period that makes the findings more reliable and appropriate, despite the heterogeneous nature of the population. Additionally, the population’s age was standardized while measuring mortality rates, which removes any variation in findings resultant from age difference within quintiles.

The principal limitation of this study is that the outcome measured is the site of death registered, which is not necessarily the same place that the patient spent most of his or her last few months receiving end of life care, which would have been more appropriate for the purpose of this study. Deprivation allocation within this study is based on the WIMD score for LSOAs, rather than socioeconomic information about individuals. However, not all people residing within a specific deprivation quintile are equally deprived and have an equal socioeconomic status.

## Conclusion

For the first time, this study allows us to look into socioeconomic deprivation and access to healthcare prevailing in Wales and their ability to influence end of life care and place of death in the region. It highlights the organization of dying in different areas across Wales and shows that large differences exist in the proportion of deaths and place of death among residents of different deprivation quintiles and that these differences are accounted for in part by provision of access to health care services and resources and in part by the level of deprivation of the geographical regions in which they are living. It also shows a broad difference between the actual and preferred place of death among the Welsh population. Despite all unfavourable health indicators for deprived regions in Wales, home deaths was high. Further investigation is required to understand the reason for the contradictory findings. It is also recommended that further research is carried out to find out the quality and cost of care in all locations that provide end of life care in Wales, because currently there is little evidence on the quality of care experienced by the patient and the family and costs for both the patient and healthcare providers in each of the six locations.

## Abbreviations

LSOA: Lower Super Output Area
ONS: Office for National Statistics
SMR: Standard Mortality Rate
WIMD: Welsh Index of Multiple Deprivation

## References

[1] Gomes B (2006). Factors influencing death at home in terminally ill patients with cancer: systematic review. BMJ.

[2] Beccaro M (2006). Actual and preferred place of death of cancer patients. Results from the Italian survey of the dying of cancer (ISDOC). J Epidemiol Community Health.

[3] Foreman LM, Hunt RW, Luke CG, Roder DM (2006). Factors predictive of preferred place of death in the general population of South Australia. Palliat Med.

[4] Higginson IJ, Sen-Gupta GJA (2000). Place of care in advanced cancer: a qualitative systematic literature review of patient preferences. J Palliat Med.

[5] Rainsford S, MacLeod R, Glasgow N (2016). Place of death in rural palliative care: a systematic review. Palliat Med.

[6] Curie M (2013). Patient choice v cost graphics.

[7] Imison C, Thompson J (2012). Older people and emergency bed use.

[8] Marmot M (2010). Fair society, healthy lives: the marmot review: strategic review of health inequalities in England post-2010.

[9] Bacon SL, Bouchard A, Loucks EB, Lavoie KL (2009). Individual-level socioeconomic status is associated with worse asthma morbidity in patients with asthma. Respir Res.

[10] Adler NE, Ostrove JM (1999). Socioeconomic status and health: what we know and what we don’t. Ann N Y Acad Sci.

[11] Szwarcwald CL, Souza-Júnior PR, Damacena GN (2010). Socioeconomic inequalities in the use of outpatient services in Brazil according to health care need: evidence from the world health survey. BMC Health Serv Res.

[12] Mackenbach JP, Stirbu I, Roskam A-JR, Schaap MM, Menvielle G, Leinsalu M, Kunst AE (2008). Socioeconomic inequalities in health in 22 European countries. N Engl J Med.

[13] Regidor, E., Martínez, D., Calle, M. E., Astasio, P., Ortega, P., & Domínguez, V. (2008). Socioeconomic patterns in the use of public and private health services and equity in health care. BMC Health Serv Res, 8(1), . doi:10.1186/1472-6963-8-183. 10.1186/1472-6963-8-183 PMC2551602 18789164

[14] Lahana E, Pappa E, Niakas D (2011). Do place of residence and ethnicity affect health services utilization? Evidence from greece. Int J Equity Health.

[15] End of Life Care Intelligence Network. (2013). Variations in place of death in England. http://www.endoflifecare-intelligence.org.uk/resources/publications/variations_in_place_of_death. Accessed 15 May 2016.

[16] Welsh Government (2015). End of life care annual report 2015.

[17] Marie Curie Cancer Care (2013). Death and dying.

[18] Office for National Statistics (2015). Lower super output area mid-year population estimates.

[19] StatsWales (2015). WIMD 2014 local authority analysis.

[20] Welsh Government (2014). Welsh index of multiple deprivation (WIMD) 2014 revised.

[21] Statistics, O. F. N (2015). Lower super output area mid-year population estimates.

[22] Office For National Statistics (2016). Methodology guide for mid-2015 UK population estimates (England and wales), June 2016.

[23] National End of Life Care Intelligence Network (2010). Variations in place of death in England inequalities or appropriate consequences of age, gender and cause of death?.

[24] Welsh Government (2013). Mortality statistics in wales 1.

[25] Cohen J, Bilsen J, Addington-Hall J, Löfmark R, Miccinesi G, Kaasa S, Deliens L (2008). Population-based study of dying in hospital in six European countries. Palliat Med.

[26] NHS Wales (2012). Interactive map.

[27] Public Health England. (2013). Deprivation and death: Variation in place and cause of http://www.endoflifecare-intelligence.org.uk/resources/publications/deprivation_and_death. Accessed 15 2016.

[28] Marie Curie cancer care (2012). . https://www.mariecurie.org.uk/globalassets/media/documents/commissioning-our-services/publications/understanding-cost-end-life-care-different-settingspdf. Accessed 28 Aug 2016.

